# Doxycycline Induces Mitophagy and Suppresses Production of Interferon-β in IPEC-J2 Cells

**DOI:** 10.3389/fcimb.2017.00021

**Published:** 2017-02-01

**Authors:** Yang Xing, Zhu Liqi, Lin Jian, Yu Qinghua, Yang Qian

**Affiliations:** ^1^Key Laboratory of Animal Physiology and Biochemistry, Ministry of Agriculture, Nanjing Agricultural UniversityNanjing, China; ^2^Department of Zoology, College of Life Sciences, Nanjing Agricultural UniversityNanjing, China

**Keywords:** doxycycline, mitophagy, IPEC-J2 cells, IFN-β, TGEV

## Abstract

Previous reports have demonstrated that the second-generation tetracycline derivative doxycycline (DOX) interrupts mitochondrial proteostasis and physiology, inhibits proliferation of many cell types, and induces apoptosis. However, the effects of DOX, which is widely used in porcine husbandry by feed, on the porcine intestinal epithelium are unclear. In this study, we demonstrated that DOX damaged mitochondrial morphology and induced the co-localization of mitochondria with autophagosomes, suggesting that DOX induces mitophagy in IPEC-J2 cells. We also found evidence that DOX increased intracellular levels of reactive oxygen species (ROS) or mitochondrial-specific ROS in a dose dependent manner. Moreover, 50 μg/ml DOX significantly decreased production of interferon-β and facilitated replication of transmissible gastroenteritis coronavirus in IPEC-J2 cells. These results demonstrated that DOX induced mitophagy and ROS production, which damaged the intestinal epithelium. As DOX is used extensively in pig husbandry, uncontrolled application poses a significant threat of viral infection, so stricter policies on its usage should be required.

## Introduction

Intestinal epithelial cells, which were originally called “mitochondria-rich” cells, are important for absorbing nutrition and mediating the immune response (Brown and Breton, 1996). Moreover, mitochondria play an important role with regard to reactive oxygen species (ROS), inducing programmed cell death and transducing stress and metabolic signals (Albers and Beal, 2000; Galluzzi et al., 2012). Therefore, mitochondrial biogenesis is tightly regulated to maintain overall cellular homeostasis (Wai and Langer, 2016). A variety of pathogens and chemicals can affect the mitochondrial dynamics of intestinal epithelial cells and destroy cell homeostasis (Novak and Mollen, 2015).

With the rapid development of the breeding industry, an increasing number of antibiotics are being used as veterinary drugs and feed additives (Du and Liu, 2012). Depending on the types and sizes of animals and the type of antibiotic, the dose of antibiotic varies from 3.0 to 220.0 g/kg of feed (McEwen and Fedorka-Cray, 2002). Doxycycline (DOX) is a second-generation tetracycline derivative used widely in pig husbandry (Zhang et al., 2015). It is reported that the concentration of DOX in swine manure that collected from different developing phases of livestock in 4 typical farms of Tianjin, China, were in the range of 8.6–59.8 mg/kg (Xian-Gang et al., 2008). In fact, very high concentration of veterinary antibiotics (up to hundreds of mg/kg) have frequently been found in animal excreta in China (Chen et al., 2012). According to the drug specifications of commercial doxycycline, the recommended usage does of DOX is in the range of 100–200 mg/kg of feed or water in China. It occupies the A-site of the bacterial 30S ribosomal subunit and blocks recruitment of aminoacyl-tRNA into the bacterial ribosome, which inhibits protein synthesis (Chopra, 1994). Given the proteobacterial origin of mitochondria, DOX also has powerful inhibitory effects on mitochondrial ribosomes and protein synthesis (Wang et al., 2015). This antibiotic can destroy mitochondrial dynamics and promotes the accumulation of dysfunctional mitochondria in cells (Moullan et al., 2015).

Mitophagy is a special selective type of autophagy that is essential for maintaining balanced mitochondrial dynamics by clearing dysfunctional or damaged mitochondria (Kim et al., 2007). Mitophagy has emerged as a regulatory mechanism that controls the antiviral innate immune response against intracellular pathogens (Deretic and Levine, 2009). Many studies have shown that mitochondrial dynamics play important roles in the interaction of molecules, such as mitochondrial antiviral signaling protein, in the downstream signaling of interferon (IFN) synthesis (Castanier et al., 2010). Evidence suggests that some antibiotics, including DOX, may negatively affect the immune response (Glette et al., 1984; Bellahsene and Forsgren, 1985; Woo et al., 1999).

However, the effects of DOX on pig intestinal epithelial cells are equivocal, and questions about whether DOX induces mitophagy in these cells remain unanswered. In this study, we evaluated the effects of DOX on the IPEC-J2 cell line, as an *in vitro* model of swine small intestine epithelium. We first found that DOX induced mitophagy rather than apoptosis in this cell line. Moreover, DOX decreased IFN-β production in IPEC-J2 cells transfected with poly (I: C). These findings suggest that use of DOX in the pig industry sabotages the antiviral innate immune response of swine intestinal epithelial cells.

## Materials and methods

### Cells, antibodies, and reagents

IPEC-J2 cell line (Guangzhou Jennio Biotech Co, Ltd., China), a non-transformed intestinal cell line originally derived from jejunal epithelia isolated from a neonatal, unsuckled piglet and maintained as a continuous culture (Rhoads et al., 1994), were propagated in high-glucose DMEM (Life Technologies, Shanghai, China) containing 10% FBS (Life Technologies, Shanghai, China), 16 mM HEPES (Life Technologies, Shanghai, China) and 100 μg/ml penicillin-streptomycin (Life Technologies, Shanghai, China) under a 5% CO_2_ atmosphere at 37°C. Cells were seeded in plastic tissue culture flasks (25 cm^2^ flasks, Corning, Shanghai, China) at a density of 2 × 10^5^/ml and passaged every 72–90 h for a maximum of 30 passages. Rabbit anti-LC3B was purchased from Beyotime Institute of Biotechnology (Haimen, China). Mouse anti-β-tubulin and HRP-conjugated secondary antibodies were purchased from Multisciences (Hangzhou, China). Chemical reagents used in this study were chloroquine (CQ) and Carbonyl cyanide 3-chlorophenylhydrazone (CCCP) purchased from Sigma-Aldrich. Rapamycin was purchased from Gene Operation (Michigan, USA). DOX and rotenone were purchased from Beyotime Institute of Biotechnology. Poly (I: C) was purchased from InvivoGen. Doxycycline is dissolved in deionized water at stock concentration of 20 mg/ml.

### Plasmids and generation of stable cells

The plasmids used in this study: pLVX-mitomCherry-IRES-EGFP-LC3B, pLVX-EGFP-LC3, PLVX-mRFP-EGFP-LC3, pLVX-mRFP-EGFP-BclxL, were kept in our laboratory, and the construction of those plasmids were described (Zhu et al., 2016). Lentiviral production was achieved through calcium phosphate transfection of four plasmids, according to the manufacturer's instructions (Wurm et al., 2001). To generate IPEC-J2/mitomcherry-EGFP-LC3B, IPEC-J2/EGFP-LC3, IPEC-J2/mRFP-EGFP-LC3, IPEC-J2/mRFP-EGFP-Bclxl stable cells, lentiviral supernatant was added to the cells with the supplement of Polybrene (8 mg/ml) at a MOI (multiplicity of infection) of 1. After 8 h infection, the cells were expanded in DMEM with 5 μg/ml puromycin for 2 weeks, and the surviving cells were maintained in medium supplemented with 2 μg/ml puromycin.

### Cell viability assay

Cell viability was determined by 3-(4, 5-dimethylthiazol-2-yl)-2, 5-diphenyl tetrazoliumbromide (MTT) assay. Cells were seeded in 96-well plate at 1000–3000 cells per well overnight. After incubated with DOX either for the indicated concentrations or time period, 10 μl MTT (5 μg/ml MTT in PBS; Sigma) was added to each well and incubated at 37°C for 2 h and then removed the supernatant. DMSO (Sigma, 100 μl per well) was used to dissolve the cell pellets. After shaking for 10 min, the absorbance was measured at a wavelength of 570 nm. All of the experiments were performed in sextuplicate, and the relative cell viability (%) was expressed as a percentage relative to the untreated control cells.

### Flow cytometry

The fluorescent probe 6-carboxy-2′, 7′-dichorodihydrofluorescein diacetate (DCFH-DA) and mitochondrial superoxide indicator MitoSox Red (Life Technologies) were used to measure the intracellular production of ROS or mitochondrial ROS (mitoROS), respectively. After 24 h treatment with DOX, cells were incubated with 10 μM DCFH-DA or 5 μM MitoSox serum-free medium for 10 min at 37°C. Afterwards, cells were harvested and resuspended in 500 μl of PBS, and DCF and MitoSox Red fluorescence were measured by FACS.

To detect the mitochondrial membrane potential (Δψ) after 24 h incubation of DOX by fluorescence. Cells were stained with 1 μg/ml Rhodamine 123 for 25 min at 37°C. After staining, cells were then washed with PBS and analyzed at FL-1 by FACS.

Hundred-Nanometer MitoTracker Green FM (total mitochondria) (Life Technologies) and 500 nM MitoTracker Red CMXRos (functional mitochondria) (Life Technologies) were introduced to monitor dysfunctional mitochondria by fluorescence. After 24 h incubation of DOX, cells were stained by MitoTracker for 25 min at 37°C, then washed with PBS and analyzed at FL-1 and FL-3 by FACS.

Apoptotic cell death was measured by Annexin V/propidium iodide (PI) staining assay (Miltenyi Biotec, Shanghai, China) according to manufacturer's instructions. In a word, cells were harvested and washed once with PBS, then resuspended in 100 μl binding buffer followed by incubation with 10 μl Annexin V per test for 20 min. Cells was washed again, suspended in 500 μl of binding buffer and 5 μl PI per test was added and immediately analyzed by flow cytometry (FACS). All data were analyzed using FlowJo software (Version 7.6.5, Tree Star Inc. Ashland, Oregon). All of the experiments were performed in three independent experiments. The relative intensity (%) was expressed as a percentage relative to the untreated control cells.

### Quantitative RT-PCR

For quantitative reverse transcription (RT)-PCR (qPCR), total cellular RNA was extracted with TRIZOL (Life Technologies), and RNA was reverse transcribed using the synthesis system (TaKaRa, Dalian, China). qPCR was performed using the Real-Time PCR system (ABI 7500, Life Technologies, USA). Gene expression from three independent experiments was calculated with the comparative Ct method and normalized to the endogenous levels of GAPDH. Primer sequences used for qPCR are as follows: NFE2L2 (Nrf2), 5′-AGCCCAGTCTTCATTGCTCC-3′ and 5′-CGTGCTAGTCTCAGCAAGGT-3′;SOD2,5′-TGCTCAGAACGGACCGAGT-3′ and 5′-AGGATGCTTTGTGAACCGGC-3′;IFNB1,5′-TGCATCCTCCAAATCGCTCT-3′ and 5′-ATTGAGGAGTCCCAGGCAAC-3′;DDX58(RIG-I),5′-CCTGGTTTAGGGACGATGAGG-3′ and 5′-TCCAAAAAGCCTCGGAACCA-3′;IFIT1,5′-ACCAGACAGGGCTTTGCTAC-3′ and 5′-CTTCTGCTTTGCTGTGGTCG-3′;GAPDH,5′-TCATCATCACTGCCCCTTCT-3′ and 5′-GTCATGAGTCCCTCCACGAT-3′.

### Western blot analysis

Cells were lysed in RIPA buffer (50 mM Tris-HCl (pH 7.4), 150 mM NaCl, 1% NP-40) containing a protease inhibitor cocktail (Yhermo Science). The protein concentration was determined. Equal amounts of protein were separated by SDS-PAGE and electrophoretically transferred onto a polyvinylidene difluoride (PVDF) membrane (Millipore, Shanghai, China). After blocking with 5% nonfat milk in Tris-buffered saline containing 0.1% Tween 20, the membrane was incubated with specific primary antibodies (1:1000), followed by incubation with appropriate horseradish peroxidase-conjugated secondary antibodies. Signals were detected using SuperSignal WestPico kit (Thermo Scientific) and subjected to Image Reader LAS-4000 imaging system (FUJIFLIM, Japan).

### Fluorescence microscopy

Cells were grown on coverslips and fixed with 4% paraformaldehyde prepared in PBS for 30 min at room temperature. After 3 washes with PBS, cells were incubated with DAPI (blue) to stain the nucleus. Then cells were observed with a Zeiss LSM710 confocal microscope (Carl Zeiss, Germany), the images were analyzed using ZEN 2012 (Blue edition) (Carl Zeiss).

### Viral infection and drug treatment

TGEV (SHXB strain) was a kind gift of Jiangsu Provincial Academy of Environmental Science (JAAS), and propagated in ST cells. IPEC-J2 cells were treated with DOX at the concentrations indicated for 24 h. Then those cells were infected with TGEV at MOI of 5 for 1 h at 37°C. Unattached viruses were removed and the cells were washed 3 times with PBS. The cells were incubated in 2% FBS medium supplemented with DOX at indicted concentrations. The samples were harvested and viral titers were determined by 50% endpoint dilution assays (50% tissue culture infective dose [TCID50]) or viral plaque assay on ST cells.

### Statistics

Data are presented as means ± SEM. Statistical analysis was performed using Statistical Program for Social Sciences (SPSS) 16.0. Significance was determined by Analysis of Variance (ANOVA). *P* < 0.05 was considered weakly significant, *P* < 0.01 significant and *P* < 0.001 highly significant.

## Results

### Effects of DOX on IPEC-J2 cell viability

**Figure 1:**
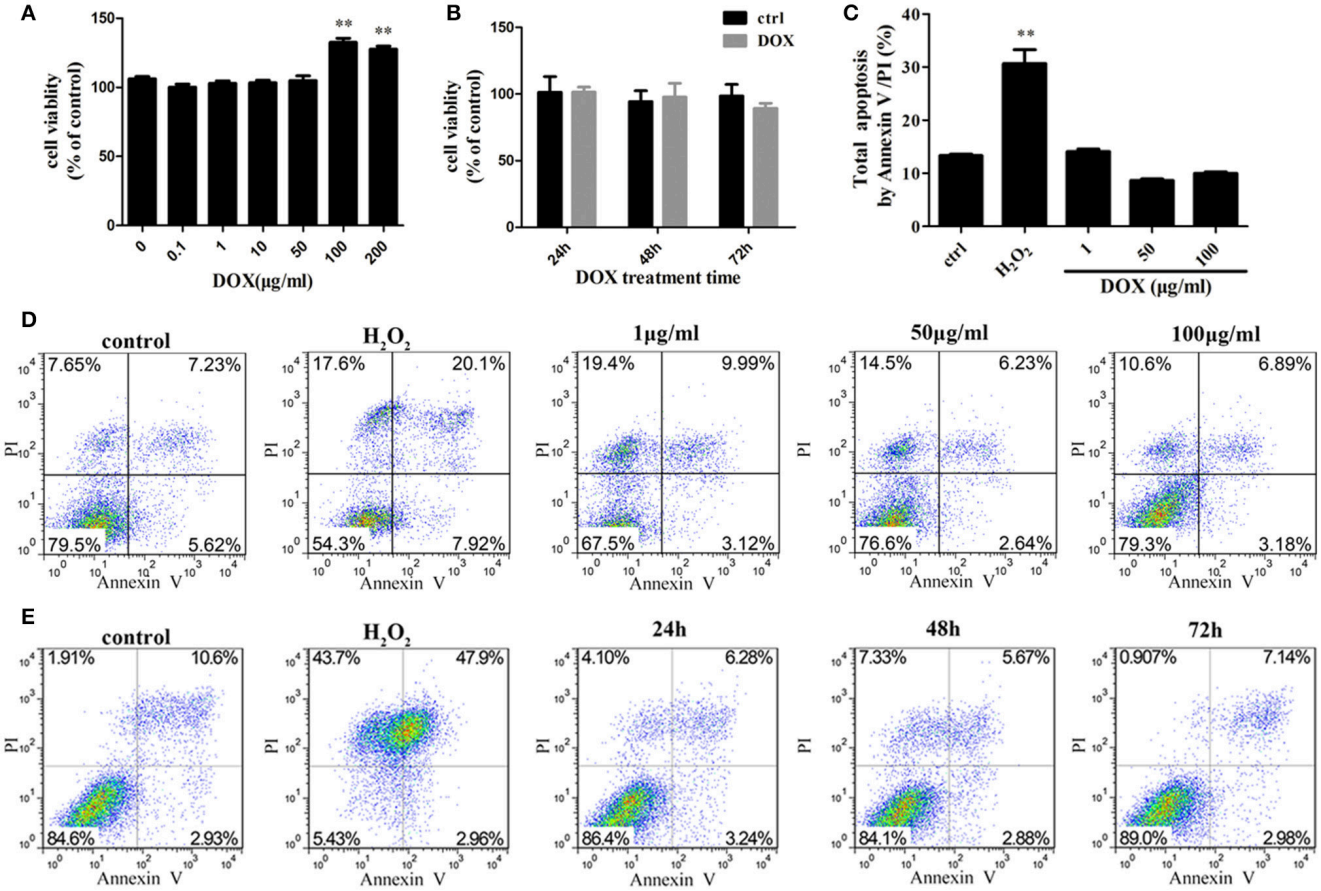
**Effects of DOX on IPEC-J2 cell viability. (A,B)** IPEC-J2 cells were seeded in 96-well plates. After (0–200 μg/ml) DOX treatment 24 h or 50 μg/ml DOX treatment 24, 48, and 72 h, cell viability was determined by MTT assay. **(C,D)** IPEC-J2 cells were treated with (1–100 μg/ml) DOX for 24 h or **(E)** treated with 50 μg/ml DOX for 24, 48, and 72 h. Cell apoptosis was analyzed by flow cytometry. Data are means ± standard deviations of three independent experiments. One-way analysis of variance; ^**^P < 0.001.

To investigate the sensitivity of IPEC-J2 cells to DOX, we determined the cytotoxic effect by testing cell viability. No inhibitory effect of DOX at therapeutic levels used in animal feed were detected in IPEC-J2 cells after a 24-h incubation (Figures 1A,B). As DOX could induce apoptosis in different type cells (Fife et al., 1997; Wu et al., 2006; Lai et al., 2007; Mouratidis et al., 2007; Yeh et al., 2007), we evaluated whether the growth inhibitory effect of DOX was associated with apoptosis or necrosis, we used a double-staining method with fluorescein isothiocyanate-conjugated AnnexinV and propidium iodide to examine apoptosis in IPEC-J2 cells. The flow cytometric analysis did not reveal any distinct apoptotic changes in DOX-treated IPEC-J2 cells after 24 h of treatment (Figures 1C,D), or in 50 μg/ml DOX-treated IPEC-J2 cells for 24, 48, and 72 h (Figure 1E). Taken together, these results verify that DOX as food additives have little effect on IPEC-J2 cells viability, and would not induce apoptosis.

### Effects of DOX on IPEC-J2 cell mitochondria

**Figure 2:**
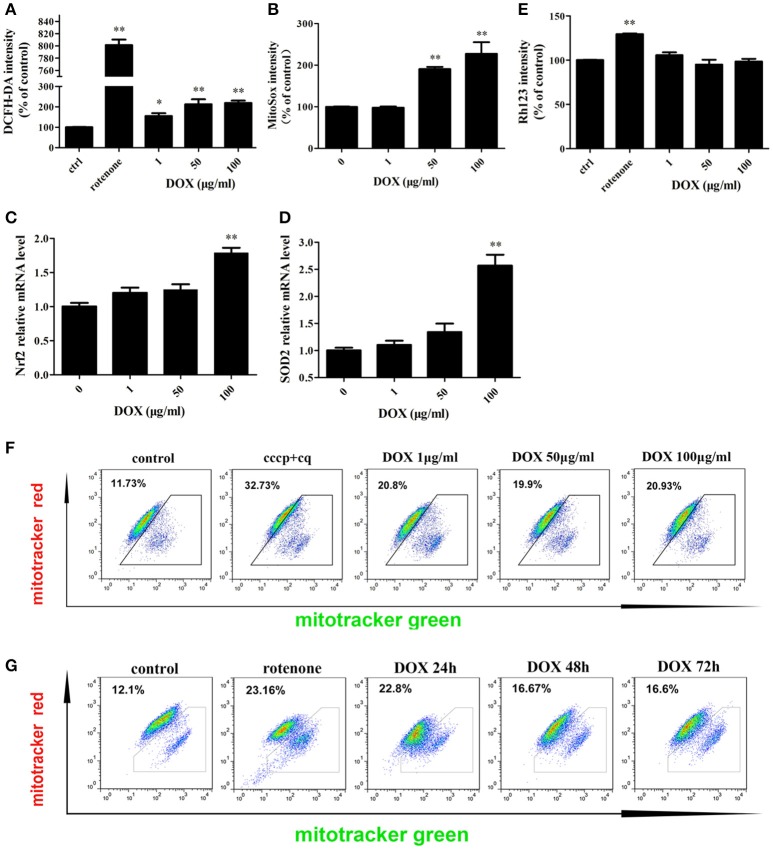
**Effects of DOX on IPEC-J2 cell mitochondria. (A,B)** IPEC-J2 cells were treated with DOX for 24 h. Rotenone (100 μM) was as a positive control. Reactive oxygen species (ROS) and mitoROS levels in cells were detected by DCFH-DA or MitoSox fluorescent probes, respectively, and quantified by flow cytometry (FACS). **(C,D)** Nrf2 and superoxide dismutase (SOD)2 mRNA expression levels were normalized relative to the control. **(E)** IPEC-J2 cells were treated as in A, harvested, stained with Rhodamine 123, and subjected to FACS to examine mitochondrial membrane potential. Mean intensities were normalized relative to the control. **(F)** IPEC-J2 cells were treated as A, **(G)** 50 μg/ml DOX treatment 24, 48, and 72 h, the cells were harvested and stained with MitoTracker Green and MitoTracker Red. Contour plots of the FACS analysis are depicted. The proportion of dysfunctional mitochondria was calculated from three independent experiments as 100% × [(green stained mitochondria) − (red-stained mitochondria)]/(green-stained mitochondria). Data are means ± standard deviations of three independent experiments. One-way analysis of variance; ^*^P < 0.01; ^**^P < 0.001.

Mitochondria are the major sites for the generation of ROS, including superoxide, hydroxyl radicals, and hydrogen peroxide (H_2_O_2_), in non-phagocytic cells. Antibiotics that induce mitochondrial dysfunction could cause oxidative damage in mammalian cells (Kalghatgi et al., 2013). DOX also affects mitochondrial morphology in cultured cells (Moullan et al., 2015). We tested whether DOX induces production of intracellular ROS in IPEC-J2 cells. DCF fluorescence intensity, which indicates the intracellular level of ROS, increased significantly after a 24-h incubation with the positive control rotenone or DOX at all concentrations examined (Figure 2A). The mitochondrial-specific ROS indicator MitoSox, which could selectively detects superoxide in mitochondria, was used to examine mitochondrial ROS levels. Once oxidized by superoxide, mitochondrial-targeted MitoSox generates red fluorescence. MitoSox fluorescence was enhanced in a dose-dependent manner (Figure 2B). We also measured the mRNA levels of Nrf2 and superoxide dismutase (SOD)2, which are involved in modulating ROS levels, to further examine oxidative stress status. According to the results, their expression were increased in a dose-dependent manner after a 24-h exposure to DOX (Figures 2C,D). We speculated that DOX could lead to the accumulation of ROS and damaged mitochondria. The mitochondrial membrane potential was assessed by Rh123, but no changes were detected (Figure 2E). We then used two types of mitochondrial-specific label to distinguish respiring (MitoTracker Red) vs. total (MitoTracker Green) mitochondria. Dysfunctional non-respiring (MitoTracker green-positive, MitoTracker red-negative) mitochondria increased dramatically after DOX treatment (Figures 2F,G). DOX could induce oxidative stress and increase the accumulation of dysfunctional mitochondria in IPEC-J2 cells.

### DOX induces complete autophagy in IPEC-J2 cells

**Figure 3:**
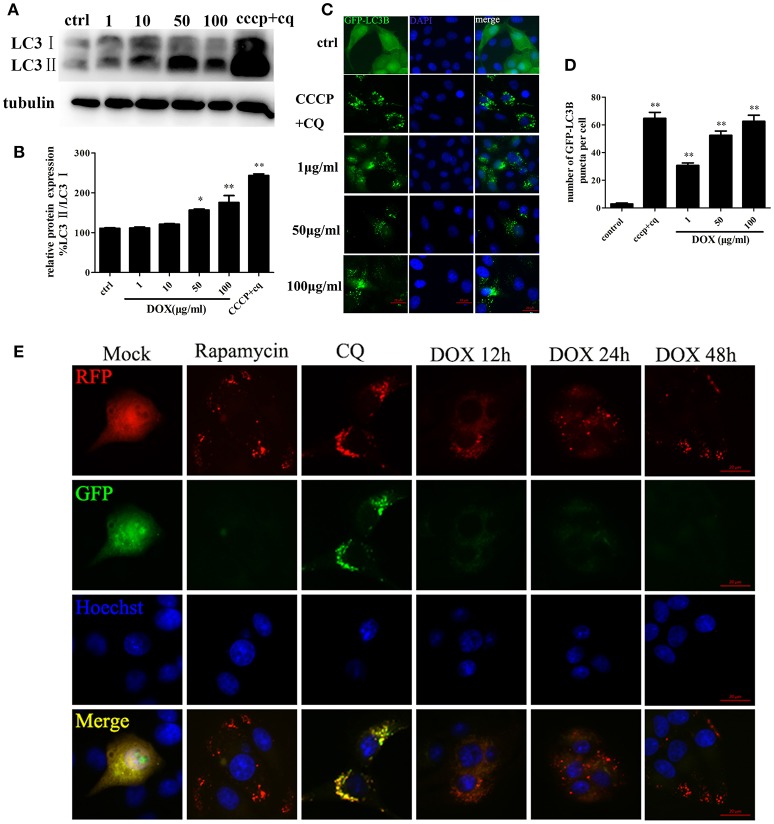
**DOX induces complete autophagy in IPEC-J2 cells. (A, B)** IPEC-J2 cells were treated with DOX, and CCCP + chloroquine (CQ) treatment was used as a positive control. LC3I, LC3II, and GAPDH expression (loading control) were analyzed by Western blot using specific antibodies. LC3II/LC3I levels normalized relative to control cells are shown. **(C,D)** IPEC-J2 cells stably expressing enhanced green fluorescent protein (EGFP)-LC3 were treated with DOX or CCCP+CQ and stained with DAPI. The number of GFP-LC3 puncta per cell was quantified. **(E)** IPEC-J2 cells stably expressing monomeric red fluorescent protein (mRFP)-EGFP-LC3 were treated with rapamycin or CQ for 12 h or treated with 50 μg/ml DOX for 12, 24, and 48 h and stained with Hoechst 33342 (blue color in the images) and observed by fluorescence microscopy. Yellow puncta (incomplete autophagic flux); red puncta (complete autophagic flux). Data are means ± standard deviations of three independent experiments. One-way analysis of variance; ^*^P < 0.01; ^**^P < 0.001.

Based on the evidence that autophagy protects cells from apoptosis and the so-called apoptosis/autophagy paradox hypothesis (González-Polo et al., 2005; Fimia and Piacentini, 2010), we determined whether DOX induces autophagy in IPEC-J2 cells. LC3 is a labeling protein found on the autophagosome membrane. Newly synthesized LC3 is diffusely distributed in the cytoplasm of mammalian cells. However, LC3I is transformed into LC3II when autophagy occurs, and the latter accumulates on autophagosome membranes. Therefore, the degree of autophagy can be determined by the LC3II: LC3I ratio (Tanida et al., 2004; Feng et al., 2013). Thus, we examined expression of the LC3 autophagosome protein in IPEC-J2 cells by Western blot analysis. The LC3II: LC3I ratio increased in a dose-dependent manner during a 24-h incubation with DOX compared with the control; the exception was the 1 μg/ml DOX group, in which the LC3II: LC3I ratio increased only slightly (Figures 3A,B). To this end, we used CCCP and the lysosomal inhibitor chloroquine (CQ) (Bjørkøy et al., 2009) as a positive-control group. Moreover, we established stable IPEC-J2 cells that expressed the green fluorescent protein (GFP)-LC3 fusion protein. DOX treatment led to the punta formation in GFP-LC3-labeled vesicles. In contrast, most cells in the control group displayed a weak and diffuse cytoplasmic GFP-LC3 fusion protein signal (Figure 3C). The number of GFP-LC3B puncta per cell is shown in Figure 3D. An increase in LC3II or GFP-LC3 vesicles can occur due to increased synthesis of autophagosomes and due to impaired autophagosome–lysosome fusion. Therefore, a simple assessment of LC3II levels or LC3-positive vesicles cannot distinguish between the two scenarios. Thus, we used the monomeric red fluorescent protein (mRFP)-GFP-LC3 tandem reporter construct to measure autophagic flux (Kimura et al., 2007). The green fluorescence of this tandem autophagosome reporter is attenuated in the acidic pH of the lysosome by lysosomal hydrolysis, whereas red fluorescence is not. Therefore, autophagosomes have both GFP and mRFP signals, whereas autolysosomes have only mRFP signals, because GFP is attenuated in the acidic lysosomal environment. RFP-LC3-labeled puncta structures were detected in IPEC-J2 cells expressing the mRFP-GFP-LC3 reporter after incubation with DOX (Figure 3E). Treatment with the autophagy inducer rapamycin also increased the number of RFP-LC3-labeled puncta structures. In contrast, treatment with CQ resulted in yellow color-labeled autophagosomes (Figure 3E). These observations indicate that DOX induced complete autophagy in IPEC-J2 cells.

### DOX induces complete mitophagy

**Figure 4:**
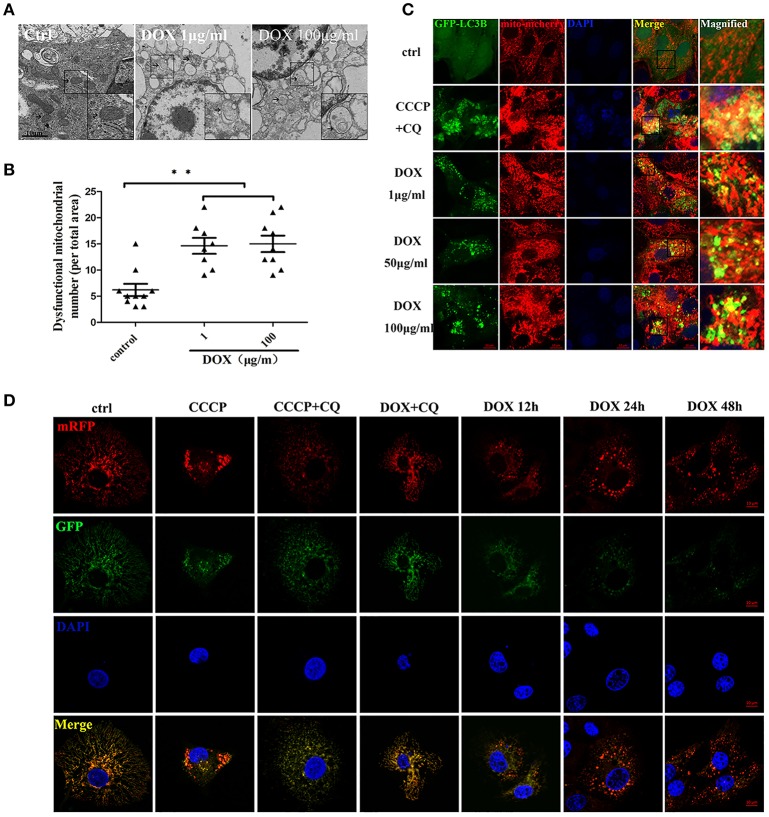
**DOX induces complete mitophagy. (A)** Transmission electron microscopic pictures after 24 h with or without DOX (1 or 100 μg/ml). Arrows at the ctrl point are normal mitochondria with clear cristae, and arrows in the DOX treatment picture indicate abnormal mitochondria without clear cristae. Images in the black box were enlarged 2.5 times and placed in the lower right corner of each picture. Scale bars = 10 μm. **(B)** Dysfunctional mitochondria number in per total area is shown. **(C)** IPEC-J2 cells were treated with DOX for 24 h or with CCCP + chloroquine (CQ) as a positive control. Cell nuclei were stained with DAPI, and fluorescence signals were visualized by confocal immunofluorescence microscopy. Green fluorescence indicates autophagosomes, and red fluorescence indicates mitochondria. Yellow fluorescence indicates co-localized autophagosomes and mitochondria. Higher-magnification images represent the regions enclosed in black squares. **(D)** IPEC-J2 cells stably expressing monomeric red fluorescent protein (mRFP)-enhanced green fluorescent protein (EGFP)-Bcl-xL were treated with CCCP, CCCP + CQ, or DOX + CQ for 24 h or treated with DOX alone for 12, 24, or 48 h. These cells were stained with DAPI and observed by confocal fluorescence microscopy. Data are means ± standard deviations of three independent experiments. One-way analysis of variance; ^**^P < 0.001.

Transmission electron microscopy (TEM) is used to confirm the double-membrane structure of autophagosomes containing undigested cytoplasm or organelles (Mizushima, 2004). We used TEM to observe whether DOX induced mitochondrial and autophagosome damage. As shown in Figure 4A, oval mitochondria in the control group had the complete crista structure. However, mitochondria became swollen and had few cristae in IPEC-J2 cells after a 24-h incubation with DOX. Double-membrane vesicles enclosing the mitochondria were also observed. Dysfunctional mitochondria number was shown (Figure 4B). We speculated that this structural change was caused by mitophagy. It has been reported that mitophagy clears the accumulation of damaged mitochondria, which is important for mitochondrial turnover (Hara et al., 2014). For further evidence, we constructed the multi-functional pLVX-EGFP-LC3B-IRES-mito-mCherry lentiviral vector and transfected it into IPEC-J2 cells to detect mitophagy induced by DOX. Obvious GFP-LC3B and mito-mCherry were clearly co-localized in IPEC-J2 cells after a 24-h DOX treatment at all concentrations examined (Figure 4C). However, the complete of mitophagic flux was unknown. For this reason, we used a sensitive dual-fluorescence reporter expressing mRFP-GFP-mito fused in-frame to a mitochondrial targeting sequence (Bcl-xL transmembrane structure) to monitor mitophagy and lysosomes for degradation. Many bright red color-labeled mitochondria were present as puncta after different incubation durations with DOX (Figure 4D). In contrast, treatment with CQ restored the expression of green fluorescence and resulted in yellow color-labeled mito in CCCP- or DOX-treated cells (Figure 4D).

### Mitophagy induced by DOX suppresses the antiviral innate immunity of IPEC-J2 cells

**Figure 5:**
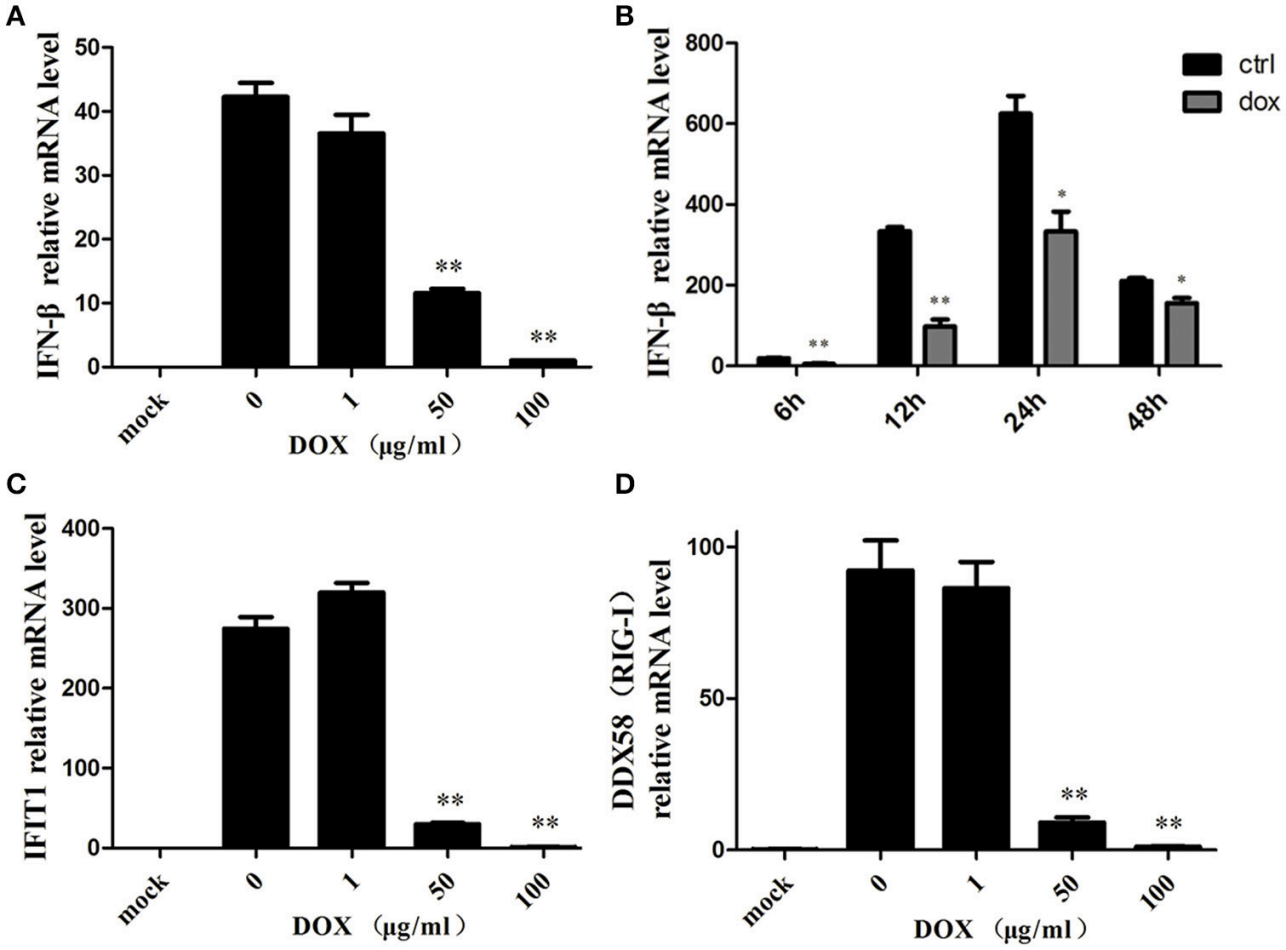
**Mitophagy induced by DOX suppresses the antiviral innate immunity of IPEC-J2 cells**. IPEC-J2 cells were pretreated with DOX for 24 h. Then, these cells were transfected with or without 500 ng/ml poly (I: C) for 24 h. RT-PCR for GAPDH was used as an internal control. RNA expression levels normalized relative to those of mock cells are shown. Relative IFN-β **(A)**, IFIT1**(C)** and DDX58 (RIG-I) **(D)** mRNA level is shown. **(B)** IPEC-J2 cells were pretreated with or without 50 μg/ml DOX for 24 h and transfected with 500 ng/ml poly (I: C) for 12, 24, and 48 h. IFN-β mRNA expression levels were normalized to the GAPDH mRNA level in each sample. Data are the means ± standard deviations of three independent experiments. One-way analysis of variance; ^*^P < 0.01; ^**^P < 0.001.

Accumulating evidence suggests that mitophagy plays a pivotal role in the antiviral innate immune response by preserving cellular homeostasis under stress (Kroemer et al., 2010); as mitochondrial fission can inhibit RLR signaling (Castanier et al., 2010), we assumed that DOX could diminish the production of the type I interferon of IPEC-J2 cells. To confirm our speculation, we examined the production of IFN-β of IPEC-J2 at the mRNA level. Consequently, following poly (I: C) transfection, the production of IFN-β was inhibited by DOX in a dose-dependent manner (Figure 5A).

IPEC-J2 cells were pretreated with DOX at a concentration of 50 μg/ml for 24 h before poly (I: C) stimulation, and this significantly reduced poly (I: C)-induced IFN at indicated times (Figure 5B). Moreover, we measured the expression of two interferon-stimulation genes (ISGs) at the mRNA level. Similar to the result for IFN-β, the expression levels of IFIT1 (Figure 5C) and DDX58 (Figure 5D) mRNAs were suppressed by DOX in a dose-dependent manner. Based on these data, we concluded that DOX had a negative effect on RIG-I-like receptor (RLR) signaling in IPEC-J2 cells.

### DOX facilitates replication of TGEV in IPEC-J2 cells

**Figure 6:**
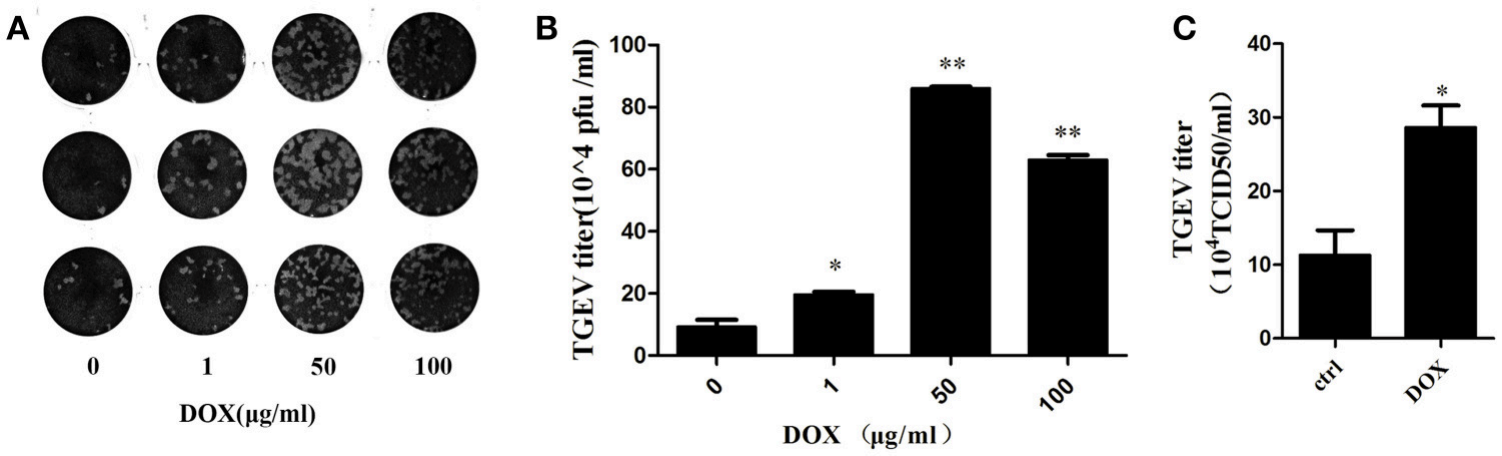
**DOX facilitates replication of TGEV in IPEC-J2 cells. (A,B)** IPEC-J2 cells were treated with DOX at the indicated concentrations for 24 h. Then, cells were infected with TGEV at a MOI of 5. The infected virus supernatants were harvested 24 hpi. Virus titer was measured by viral plaque assay. **(C)** The cell culture supernatants were harvested 24 h post-infection (hpi) and assayed for the production of infectious virus by TCID50 assay on ST cells. Each data point represents the average titer derived from two independent TCID50 assays. Error bars represent standard errors. Data represent means ± standard deviations of three independent experiments. One-way analysis of variance; ^*^P < 0.01; ^**^P < 0.001.

We introduced transmissible gastroenteritis virus (TGEV), an *Alphacoronavirus* that causes clinical watery diarrhea, dehydration, and vomiting, to piglets <2 weeks old to elucidate whether DOX contributes to replication of TGEV by suppressing IFN-β. The TGEV titers were quantified using a viral plaque assay after 24 h of TGEV infection in DOX-pretreated IPEC-J2 cells. The viral plaque formation clearly showed that DOX increased TGEV replication significantly (Figures 6A,B). Upon the infection of cells exposed to DOX, TGEV titers were also quantified by TCID50 DOX pretreatment increased the viral progeny yield in TGEV-infected IPEC-J2 cells (Figure 6C). DOX clearly suppressed the antiviral innate immunity of IPEC-J2 cells and facilitated the replication of TGEV in IPEC-J2 cells.

## Discussion

The use of antibiotics has increased dramatically in recent years but is now forbidden for promoting animal growth in the European Union, because of the development of antibiotic-resistant bacteria (Gilbert, 2011; Du and Liu, 2012). However, DOX remains the most widely used antibiotic as frontline therapy against bacterial infection (Authority and Authority, 2015). Due to the similarity of bacterial and mitochondrial ribosomes, DOX exert effects in host cells (Kroon et al., 1984; Ryan et al., 1995). The effects of DOX related to the mitochondria of individual organ systems differ because of different energy demands (Peters et al., 2011). It was reported that plasma DOX concentrations could be 1 μg/ml after pigs were administered with DOX (Schmidt et al., 2009). The gastrointestinal tract is the first target for the potential effects of doxycycline. The gastrointestinal tract can be exposed to high concentrations of DOX after ingestion of feed or drinking water with 100–200 mg/kg doxycycline. The concentration of DOX used in the individual applications can be 100 μg/ml in intestinal lumen. Therefore, we selected three DOX doses in this study: 1, 50 and 100 (μg/ml). Our results demonstrate that DOX induced mitophagy in IPEC-J2 cells, consequently decreasing IFN-β production in IPEC-J2 cells transfected with poly (I: C).

Previous studies have reported that DOX inhibits the proliferation of various cell lines (Lokeshwar et al., 2002; Wu et al., 2006; Ahler et al., 2013). In our study, DOX treated 24 h would not inhibit cell viability by the MTT assay. We observed a high dose of DOX enhanced cell viability, it might be due to that mitochondrial mass could affect the result of MTT assay (Gerlier and Thomasset, 1986). DOX inhibits oxidative phosphorylation, increases the level of ROS, and reduces ATP synthesis through its mitochondrial actions (Kalghatgi et al., 2013). Our results show that DOX increased the level of ROS or mitoROS in a dose dependent manner and induced oxidative stress in IPEC-J2 cells. The mitochondrial membrane potential of IPEC-J2 cells was not affected by DOX (Figure 2), albeit three studies have reported that DOX decreases mitochondrial membrane potential (Sourdeval et al., 2006). DOX provides a balance between mitochondrial fission and the accumulation of fragmented mitochondria (Moullan et al., 2015). The number of dysfunctional mitochondria increased significantly in IPEC-J2 cells after DOX incubation (Figure 2). We can observe that the number of dysfunctional mitochondria for both 1 and 100 ug/ml of DOX is similar. It might be due to the fact that 100 ug/ml DOX can induce significantly mitophagy to maintain relative mitochondrial dynamics by removing damaged mitochondria. A review reported that six of seven studies support the apoptotic action of DOX, whereas one study showed an anti-apoptotic effect (Sagar et al., 2010). Unexpectedly, we found that DOX increased the number of dysfunctional mitochondria without apoptosis.

The important role of mitochondria in the intrinsic apoptosis pathway led us to postulate that DOX might induce mitophagy in IPEC-J2 cells to inhibit apoptosis by removing damaged mitochondria. Indeed, mitophagy induced by a TGEV infection counteracts apoptosis in IPEC-J2 cells, and inhibiting autophagy enhances apoptosis in Hela cells infected with MV-Edm (Richetta et al., 2013). A few viruses suppress apoptosis by mitophagy during an infection (Kim et al., 2013a,b, 2014; Meng et al., 2014). Our experimental results first indicate that DOX could induce complete mitophagy in IPEC-J2 cells (Figure 4).

Mitochondrial dynamics regulate antiviral innate immunity (Castanier et al., 2010). Replication of hepatitis C virus (HCV) increases in response to Parkin-mediated mitophagy (Kim et al., 2013a), and HBV favors its own replication by promoting bulk autophagy (Tang et al., 2009; Sir et al., 2010; Li et al., 2011). The late stages of mitophagy must be completed to attenuate RLR signaling (Xia et al., 2014). Our data suggest that DOX decreased IFN-β production in IPEC-J2 cells transfected with poly (I: C) (Figure 5). As a result, DOX promoted replication of TGEV, which is a single-stranded RNA virus (Figure 6). We also provide evidence for the effects of DOX on IPEC-J2 cells. DOX induced mitophagy to mitigate the antiviral innate immune response, which might contribute to the replication of TGEV in intestinal epithelial cells. These results suggest caution to prevent the abuse of DOX in pig husbandry.

## Author contributions

YX: study conception and design, performance of the experiments, data analysis and interpretation, manuscript writing; ZL: performance of the experiments, data analysis and interpretation; LJ and YuQ: data analysis and revise this manuscript; YaQ: study conception and design, financial support, administrative support, manuscript writing, final approval of the manuscript.

### Conflict of interest statement

The authors declare that the research was conducted in the absence of any commercial or financial relationships that could be construed as a potential conflict of interest.
